# An innovative technique for the extraction and stability of polyphenols using high voltage electrical discharge: HVED-Assisted Extraction of Polyphenols

**DOI:** 10.1016/j.crfs.2024.100928

**Published:** 2024-11-16

**Authors:** Leila Abbaspour, Nazila Ghareaghajlou, Mohammad Reza Afshar Mogaddam, Zahra Ghasempour

**Affiliations:** a Students Research Committee, Department of Food Science and Technology, Faculty of Nutrition and Food Sciences, Tabriz University of Medical Sciences, Tabriz, Iran; b Food and Drug Safety Research Center, Tabriz University of Medical Sciences, Tabriz, Iran; c Nutrition Research Center, Department of Food Science and Technology, Faculty of Nutrition and Food Sciences, Tabriz University of Medical Sciences, Tabriz, Iran

**Keywords:** Degradation, Dielectric breakdown, HVED, Mechanism, Phenolic compounds

## Abstract

Polyphenols are the main group of phytochemicals with several biological activities. Due to the adverse effects of conventional solvent extraction methods, innovative extraction techniques have been used as alternatives to overcome these problems. High voltage electric discharge (HVED) is an eco-friendly innovative extraction technique based on the phenomenon of electrical breakdown in water. This technique induces physical and chemical processes, leading to product fragmentation, cellular damage, and liberation of bioactive compounds. HVED treatment can extract polyphenols at lower temperatures and shorter times than the conventional solvent extraction methods. This review summarizes the effect of HVED processing parameters on the recovery and stability of polyphenols from plant sources. Hydroethanolic solutions improve the HVED-assisted extraction of polyphenols compared to water. Moreover, acidic solvents are suitable for the high recovery and protection of polyphenols during electric discharges. This study revealed the efficacy of the HVED technique in extracting polyphenols for their utilization in the food and pharmaceutical industries.

## Highlights

• High voltage electrical discharge (HVED) relies on the electric breakdown in water.
• Adding ethanol improves the aqueous extraction of polyphenols using HVED.
• The maximum polyphenol content is achieved at acidic or basic conditions.
• Acidification has a protective effect on polyphenols during the HVED treatment.
• HVED leads to high efficiency, short extraction time, and low energy usage.

## Graphical abstract

**Figure:**
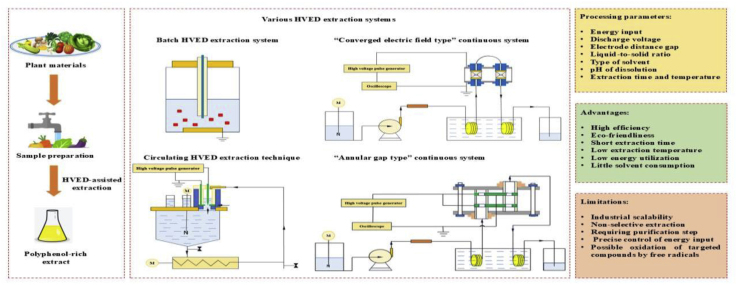

## 1 Introduction

**Fig. 1:**
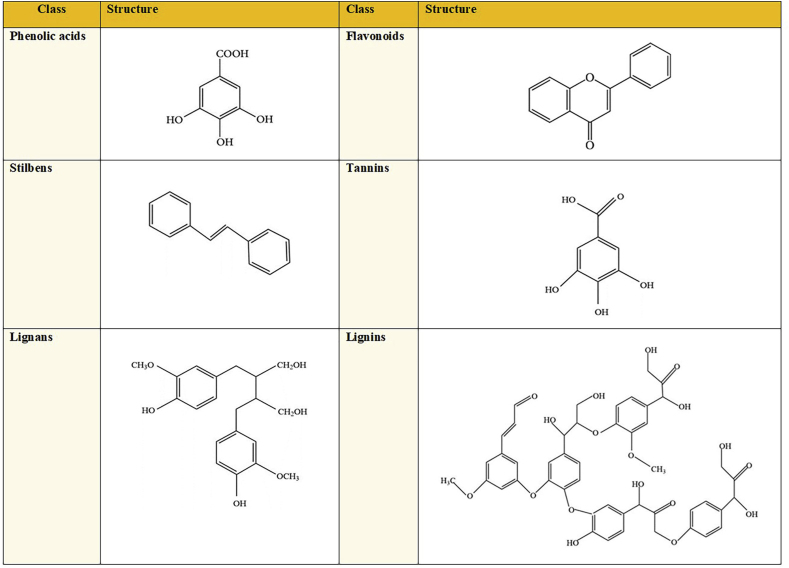
The major structure of polyphenol derivatives.

Polyphenols are the main group of phytochemicals in plant-based foods (Nayak et al., 2015). The basic structure of phenolics consists of a benzene ring linked to free or involved hydroxyl groups (Lund, 2021). These compounds are divided into derivatives, including phenolic acids, flavonoids, stilbenes, tannins, lignans, and lignins (Lund, 2021; Nayak et al., 2015) (Fig. 1). Antioxidant (Khalifa et al., 2023; Pandey et al., 2020), antimicrobial (Dalal et al., 2023; Grobelna et al., 2019a; Kalisz et al., 2020), antihyperglycemic (Choudhary and Tahir, 2023; Grobelna et al., 2020), anti-diabetic (Abduallah et al., 2023; Grobelna et al., 2020), anti-cancer (Pandey et al., 2020), anti-inflammatory (Grobelna et al., 2019a; Pandey et al., 2020), anti-allergic (Abd Rani et al., 2019), nephroprotective (Turan et al., 2023), gastroprotective (Grobelna et al., 2019b), and hepatoprotective (Saleh et al., 2023) properties are some of the reported health-promoting advantages of polyphenols in many *in vivo* and *in vitro* studies. Polyphenols are derived from plant sources, such as fruits, vegetables, and grains (Athanasiadis et al., 2023). However, the existence of these substances within the plant cell vacuoles and lipoproteins bilayers makes their recovery difficult (Coelho et al., 2020).

Conventional extraction methods, such as soaking, maceration, percolation, and Soxhlet extraction are commonly applied for the recovery of polyphenols (Athanasiadis et al., 2023). However, these methods are associated with several disadvantages, including high solvent utilization, a long extraction time, low extraction efficiency, and thermal degradation of bioactive compounds (Ranjha et al., 2021). Moreover, the toxicity, volatility, and flammability of some of the employed organic solvents, make them unfavorable for consumer's health and the environment (Foroutani et al., 2023). In this regard, it is necessary to use innovative extraction techniques to overcome the limitations of conventional solvent extraction methods (Ghareaghajlou et al., 2021). Innovative extraction techniques such as high hydrostatic pressure (HHP) (Grassino et al., 2020), ultrasounds (US) (Lončarić et al., 2020), and pulsed electric fields (PEF) (Rajha et al., 2019) have been applied to extract polyphenols. High voltage electrical discharge (HVED) is also an environmentally friendly innovative extraction technique that can be used as a pretreatment to facilitate the recovery of phenolics during the subsequent diffusion process (El Kantar et al., 2019; Shahram and Dinani, 2019). Moreover, it can decrease the required time, temperature, and ethanol content in the diffusion process compared to the conventional solvent extraction methods (Li et al., 2019; Žuntar et al., 2019). Along with increasing the extraction efficiency of polyphenols, HVED may affect the stability of these substances, which is crucial for their further application as natural antioxidants, preservatives, and bioactive ingredients in the pharmaceutical, food, and cosmetic industries (Hejazi et al., 2022; Žuntar et al., 2019). This technique can also increase the shelf life of food products through the inactivation of microorganisms (Tiban et al., 2022).

HVED allows the direct energy release into an aqueous solvent through the formed plasma channel between two submerged electrodes (Banožić et al., 2021). Two kinds of processes may participate in the formation of a plasma channel. While the first process relies on developing a gaseous phase, in the second one the breakdown is caused by the ionization of the solvent (Boussetta and Vorobiev, 2014). This technique provides a vast range of current (10^3^–10^4^ A), voltage (10^3^–10^4^ V), and frequency (10^−2^ –10^−3^ Hz) (Wang et al., 2023). HVED technology has been used for the extraction of oil from sesame seeds (Sarkis et al., 2015b), hemicelluloses from spruce (Chadni et al., 2019), lignans from flaxseed cake (Boussetta et al., 2013b), flavonoids from peanut shells (Yan et al., 2018), pectin from sugar beet pulp (Almohammed et al., 2017), and polyphenols from oregano (Nutrizio et al., 2022). To the best of our knowledge, no study has precisely reviewed the impact of HVED treatment on total polyphenol content (TPC). Therefore, this study aimed to investigate the effect of HVED processing parameters on the extractability and stability of polyphenols. Additionally, it reviews various HVED extraction systems and compares this technique with PEF and US novel extraction technologies. The future aspects and challenges of the HVED technique are also discussed at the end of the study.

## 2 Mechanism of HVED treatment

**Fig. 2:**
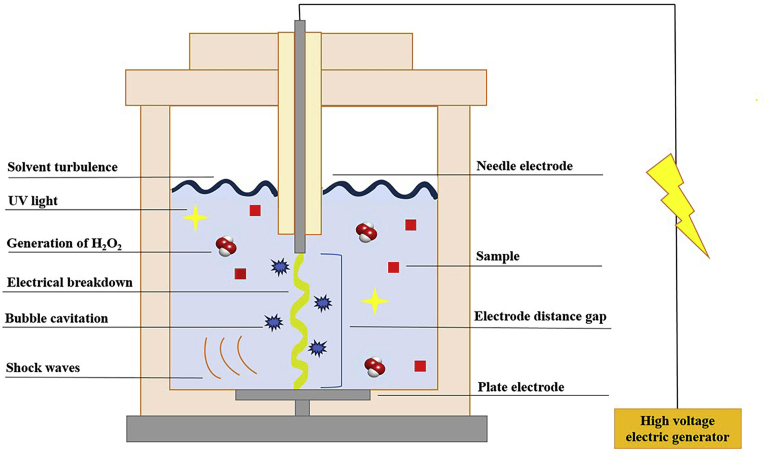
Physical and chemical processes during the HVED treatment.

The HVED technique is based on the electrical breakdown in water, including streamer (pre-breakdown phase) and arc (breakdown phase) discharge processes (Li et al., 2019; Sarkis et al., 2015a). When using a high voltage between electrodes, the electrons are accelerated and thus, get adequate energy to excite water molecules. Subsequently, an avalanche of electrons called streamer and gaseous cavities are formed (Boussetta et al., 2009a; Brianceau et al., 2016a, Brianceau et al., 2016b). If the electric field is intense enough, the streamer propagates from the positive needle electrode to the negative grounded one (Rajha et al., 2015b). At this moment, the released energy creates an electric arc between the electrodes and an electrical breakdown occurs (Hernández-Corroto et al., 2022). The induced physical and chemical processes during the electrical breakdown cause cellular damage and thus, increased recovery of intracellular compounds compared to conventional solvents (Deng et al., 2018). While physical processes include UV light emission, shock wave propagation, severe solvent turbulence, and cavitation bubbles explosion, chemical processes contain the formation of free radicals, hydrogen peroxide, and ozone (Fig. 2) (Boussetta et al., 2013a; Xi et al., 2017). It is worth mentioning that, UV light can inactivate cells by damaging the DNA and reactive radicals may cause cell oxidation. Moreover, shock waves, bubble cavitation, and solvent turbulence could lead to product fragmentation and the mechanical disruption of cells (Rajha et al., 2015a; Žuntar et al., 2019). Indeed, the combined impact of shock waves and cavitation bubbles increases the degradation and destabilization of cell walls (Chadni et al., 2019).

## 3 Types of HVED extraction systems

Generally, the HVED extraction systems can be categorized into batch, continuous, and circulating systems. Although the mechanisms of these three techniques are the same, they may have structural differences in the related devices such as electrodes (Li et al., 2019). In the following section, an overview of different HVED extraction systems has been provided.

### 3.1 Batch HVED extraction technique

**Fig. 3:**
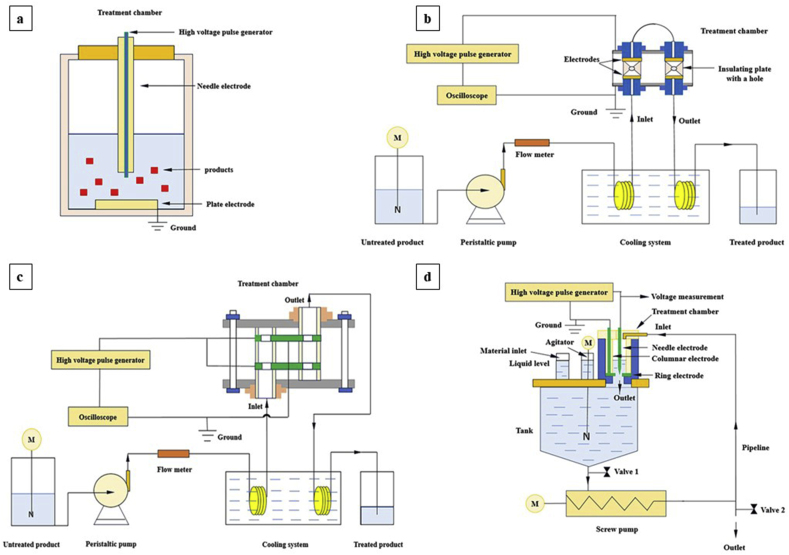
Schematics of batch (a), “converged electric field type” continuous (b), “annular gap type” continuous (c), and circulating (d) HVED extraction systems.

Several bioactive compounds have been recovered with the batch HVED extraction system in laboratory and pilot scales (Boussetta et al., 2012a). In this system, independent processes occur in a batch treatment chamber (Chemat et al., 2020). The needle-plate electrode with a positive voltage utilized on the needle is commonly applied in the batch extraction system (Fig. 3a). When the employed voltage is high enough, the high-intensity electric field is concentrated at the needle electrode, and thus, electrical discharges happen in the water (Jha and Sit, 2022). The batch HVED extraction system involves three steps: sample preparation, HVED treatment, and diffusion. First, the raw material is cleaned, dried, sieved, and stored. Second, it is mixed with solvent, and processed in the HVED treatment chamber. Third, more solvent is added to increase the diffusivity of bioactive compounds into the solvent, followed by centrifugation to isolate the supernatant for further analysis (Li et al., 2019).

### 3.2 Continuous HVED extraction technique

The separation of HVED treatment and diffusion steps in the batch HVED extraction system prolongs the extraction time. In this regard, a continuous treatment chamber is more suitable for the industrial application of HVED-assisted extraction (Li et al., 2019). This technique is divided into two types of “converged electric field type” and “annular gap type” continuous systems (Mikhaylin, 2023). The continuous HVED extraction system contains three parts: sample preparation, HVED treatment coupled with diffusion, and collection. First, a combination of the raw material and solvent is pumped into the system. It is worth mentioning that the flow rates of the materials should be adjusted to prevent clogging in the extraction system. Finally, the HVED-treated samples are passed through filter paper and centrifuged (Li et al., 2019).

#### 3.2.1 “Converged electric field type” continuous technique

Unlike the batch HVED extraction system, there is no needle-plate electrode in the treatment chamber of this extraction system. Since this system contains a pair of parallel disc mesh electrodes, the obtained uniform electric field leads to the difficult formation of electric discharge. To solve this problem, an insulating plate with a small hole is set up between the electrodes (Fig. 3b). This structure generates a more intense local electric field in the hole than in other parts. Thus, applying a high voltage facilitates the formation of discharges (Li et al., 2019).

#### 3.2.2 “Annular gap type” continuous technique

This extraction system has been designed to solve the defect of the low flow rate of the material in the “converged electric field type” continuous system (Yan et al., 2018). The applied electrodes in the “annular gap type” continuous system are neither the needle-plate nor the parallel electrodes (Li et al., 2019). This system consists of a pair of annular plate electrodes (Chemat et al., 2020) (Fig. 3c). Applying a high voltage on the two external electrodes of this system generates intense electric fields in the gap between the external and inner grounded electrodes, thus, the electric discharge forms in the gap. Since the gap width between the electrodes of the “annular gap type” system is larger than the hole in the “converged electric field type” continuous system, the processing capacity of this system is improved and the clogging is inhibited (Li et al., 2019).

### 3.3 Circulating HVED extraction technique

This system was designed to increase the processing capacity and also, achieve a higher extraction efficiency. The electrodes of the circulating HVED extraction system consist of a needle and a grounded ring. The point of the needle is located in the center of the ring (Fig. 3d) (Chemat et al., 2020). When applying a high voltage on the needle, the high-intensity electric field in the bore generates high voltage electric discharge (Li et al., 2019). Like continuous HVED extraction techniques, this system consists of three parts: sample preparation, HVED treatment coupled with diffusion, and collection. At first, a mixture of the raw material and solvent is entered into the treatment chamber through the material inlet. During the HVED treatment, the material inlet and outlet are closed and the diffusion of the bioactive compounds from the flowing material is obtained. Finally, the outlet is opened and the obtained suspension is centrifuged (Li et al., 2019).

## 4 Factors influencing the HVED-assisted extraction of polyphenols

Various HVED electrical parameters (energy input, discharge voltage, and electrode distance gap), and extraction parameters (liquid-to-solid ratio, type of solvent, pH of dissolution, extraction time, and extraction temperature) may affect the extractability of polyphenols. The HVED-assisted extraction of polyphenols from different food materials is also listed in Table 1.

**Table 1:** HVED-assisted extraction of polyphenols from different food materials.

Category	Source	Applied solvent		Liquid-to-solid ratio		Extraction conditions	Results		References
		During HVED treatment	In the diffusion process	During HVED treatment	In the diffusion process		Optimum conditions	TPC concentration	
Fruits, vegetables, and their by-products	Grape stems	Distilled water	Ethanolic solutions of 0–50% (v/v)	7.5	15	- At an electrode distance gap of 5 mm -Energy inputs of 0–188 kJ/kg -Pulse numbers of 0–400 -High voltages of 40 kV-10 kA -Temperature of 20 °C -Treatment time of 0–4 ms -pH values of 2.5–8.5	Using a treatment time of 4 ms, 50% ethanol at pH = 2.5	6.6 ± 0.2 g GAE/100 g RM	(Brianceau et al., 2016a, Brianceau et al., 2016b)
	Grape seeds	Distilled water	–	5	–	-At an electrode distance gap of 10 mm -High voltages of 40 kV-10 kA -Discharge numbers of 100, 300, 500, 600, 800, and 1000 -Temperature of 50 °C	Using a discharge number of 300	8.3 g GAE/100 DM	Liu et al. (2011)
		Distilled water	–	40	–	-At electrode distance gaps of 1–3 cm -Energy inputs of 0–188 kJ/kg -Pulse numbers of 1800 -High voltages of 60 kV-1 kA -At room temperature -Pulse duration of 0.8 μs -Treatment time of 15 min	Using an energy input of 16 kJ/kg	5000 mg/100 g DM	Boussetta et al. (2013a)
	Grape Vine shoots	Distilled water	–	20	–	At an electrode distance gap of 5 mm -Energy inputs of 101.6, 203.2, 304.8, 406.3, 507.9 and 609.5 kJ/kg -Discharge numbers of 200, 400, 600, 800, 1000, and 1200 -Discharge duration of 10 μs -High voltages of 40 kV-10 kA -Temperature of 50 °C	Using an energy input of 101.6 kJ/kg	140 mg/L	Rajha et al. (2015b)
	Grape pomace	Distilled water	10–30% (v/v) ethanolic solution	2, 3, 5, 10, and 20	–	-At electrode distance gaps of 3, 5, and 10 mm -High voltages of 40 kV-10 kA -Energy inputs of 0–800 kJ/kg -Discharge numbers of 0–1000 -Ethanolic solutions of 10–30% -Temperatures of 20, 30, 40, and 60 °C -Diffusion time up to 1 h	Using energy input of about 80 kJ/kg, electrode distance gap of 5 mm, followed by extraction with 30% ethanolic solution at 60 °C for 30 min	2.8 ± 0.4 g GAE/100g DM	Boussetta et al. (2011)
		Distilled water	–	3	–	-At an electrode distance gap of 5 mm -High voltages of 40 kV–10 kA -Discharge number of 80 -Pulse duration of 10 μs -Diffusion time of 1 h -Temperatures of 20, 40 and 60 °C	Using a temperature of 60 °C	≈0.8%	Boussetta et al. (2009a)
		Distilled water	–	5	–	At the laboratory scale: -Consisting of a 200 nF pulsed high-voltage power supply, and a high voltage pulse generator producing 40 kV–10 kA discharges -Using a total product mass of 0.3 kg, the inter-electrode space of 5 mm, temperature of 20 °C, and electrical discharges of up to 1000 pulses At the pilot scale: -Composed of a 200 or 5000 nF capacitor charging with a maximum voltage of 40 kV -Using a total product mass of 7.5 kg, frequency of 100 MHz, inter-electrode space of 5 mm, temperature of 20 °C, and electrical discharges of up to 1000 pulses	At the laboratory scale: Energy values of 213 and 53 kJ/kg for stems and skins, respectively At the pilot scale: Energy values of 400 and 133 kJ/kg for stems and skins, respectively	At the laboratory scale: -≈ 400 and 500 mg GAE/L for stems and skins, respectively At the pilot scale: ≈200 and 400 mg GAE/L for stems and skins, respectively	Boussetta et al. (2012b)
		Ethanolic solutions of 10, 20, 30, and 40% (v/v)	–	40	–	-At electrode distance gaps of 3, 4, 5, and 6 mm -Discharge voltages of 0–40 kV -Liquid-to-solid ratios of 30, 40, 50, and 60 mL/g -Flow rates of 30, 40, 50, and 60 mL/min	Using an electrode distance gap of 4 mm, discharge voltage of 12 kV, ethanolic solution of 30%, liquid-to-solid ratio of 40 mL/g, and flow rate of 50 mL/min	6.78 ± 0.17%	Deng et al. (2019)
	Grape by-product	50% (v/v) ethanol acidified with 1% (v/v) HCl, and 50% (v/v) methanol acidified with 1% (v/v) HCl	–	50	–	-At an electrode distance gap of 5 mm -Discharge voltage of 30 Kv -Electric field intensity of 60 kV/cm -Frequencies of 20, 50, and 100 Hz -Extraction times of 5, 10, and 15 min -Temperature of 25 °C	Using an energy input of 22.27 kJ/kg, frequency of 100 Hz with the solvent of acidified 50% ethanol for 15 min	3023.57 mg GAE/100 g DM	Lončarić et al. (2020)
	Pomegranate peels	Distilled water	–	20–50	–	-At electrode distance gaps of 2–5 mm -High voltages of 20 kV–10 kA -Flow rates of 8–14 mL/min -Pulse duration of 2 μs -Extraction duration of 22–39 min	Using an electrode distance gap of 3.15 mm, flow rate of 12 mL/min, liquid-to-solid ratio of 35 mL/g, and extraction duration of 26 min	196.7 ± 6.4 mg/g	Xi et al. (2017)
		Hot water	–	0.1	–	-At an electrode distance gap of 40 mm -Energy inputs of 90–100 kJ/kg -Maximal voltage of 40 kV -Total extraction time of 7 min -Temperature of 50 °C	–	46 ± 0.5 mg GAE/g DM	Rajha et al. (2019)
	Grapefruit peels	Distilled water	- DESs of Lactic acid-choline chloride, Lactic acid-sodium acetate, Lactic acid-glycine, Lactic acid-ammonium acetate, Choline chloride-tartaric acid, Lactic acid- glucose - Aqueous glycerol solutions of 10, 20, and 30% (w/v) −50% ethanol	9	10	-At an electrode distance gap of 5 mm -High voltages of 40 kV-10 kA -Energy input of 7.27–218 kJ/kg -Pulses numbers of 10–300 -Temperature of 50 °C -Time of 60 min	Using energy input of 72 kJ/kg, treatment time of 5 ms, followed by extraction with solvents of 20% aqueous glycerol, and DES of Lactic acid-glucose	–	El Kantar et al. (2019)
	Red onion peels	65% (v/v) ethanol	–	30–60	–	-At an electrode distance gap of 5 mm -Energy inputs of 12.5–50 J/mL -High voltages of 0–50 kV -Temperature of 22–25 °C -Pulse numbers of 0–100	Using an energy input of 50 J/mL and the liquid-to-solid ratio of 54	739.4 mg/L	Hejazi et al. (2022)
Oilseed crops and their by-products	Olive kernel	Distilled water	0–50 % (v/v) ethanolic solutions	5	10	-At an electrode distance gap of 5 mm -Discharges of 40 kV-10 kA -Temperature of 20 °C -Energy inputs of 0–109 kJ/kg -At pH values of 2.5–12 -Extraction time of 20 min	Using energy input of 66 kJ/kg at pH 2.5, followed by extraction with 49% ethanolic solution	626.6 mg GAE/L	Roselló-Soto et al. (2015)
	Olive leaves extracts (*Olea europaea* L.)	Distilled water and ethanolic solutions (0, 25 and 50% (v/v))	–	50	–	-At an electrode distance gap of 15 mm -Discharge voltages of 15 and 20 kV for argon gas -Discharge voltages of 20 and 25 kV for nitrogen -Maximum current of 30 mA -Pulse width of 400 μs -Treatment times of 3 and 9 min -Temperature of 22 °C	Using a discharge voltage of 20 kV for argon gas, 50% ethanolic solution, and a treatment time of 9 min	65.99 ± 0.06 mg GAE/g	Žuntar et al. (2019)
	Rapeseed and rapeseed press-cake	Distilled water	–	5–20	–	-At an electrode distance gap of 0.5 cm -Energy inputs of 0–400 kJ/kg -High voltages of 40 kV-10 kA -Pulse numbers of 1–1000 -Temperature of 20 °C	Using a liquid-to-solid ratio of 5, and energy inputs of 240 and 80 kJ/kg for rapeseeds and press-cake, respectively	-≈ 600 mg GAE/100 g for rapeseeds −559.17 mg GAE/100 g for press-cake	(Barba et al., 2015a, Barba et al., 2015b)
	Sesame cake	Distilled water	10, 30, and 50% (v/v) ethanol	8.5	20	-At an electrode distance gap of 5 mm -Treatment time of 1–7ms -Energy inputs of 0–291 kJ/kg -Temperatures of 20, 40 and 60 °C	Using energy input of 83 kJ/kg, followed by extraction with 10% ethanolic solution at 60 °C	295 mg GAE/100 g	Sarkis et al. (2015a)
	Flaxseed cake	Distilled water	0–25% ethanolic solutions	8.5	17	-At an electrode distance gap of 5 mm -High voltages of 40 kV–10 kA -Pulse numbers of 1–1000 -HVED treatment time of 1–10 ms -Diffusion time of 0–80 min -Temperatures of 20, 40, and 60 °C	Using HVED treatment time of 5 ms, ethanolic solution of 25%, diffusion time of 80 min, and temperature of 60 °C	≈5 mg/g	Boussetta et al. (2013b)
Coffee by-products	Spent coffee grounds (SCG)	Distilled water and ethanolic solutions (12, 24, and 36% (v/v))	–	30, 40, 50, and 60	–	-At flow rates of 67, 133, 200, and 267 mL/min -Discharge voltages of 8, 11, 14, and 17 kV -Extraction times of 10, 15, 20, and 25 min	Using a flow rate of 200 mL/min, discharge voltage of 11 kV, 24% ethanolic solution, and extraction time of 20 min	59.83 ± 1.53 mg/g	Deng et al. (2018)
Aromatic herbs	Oregano	Distilled water, 25%, and 50% (v/v) aqueous ethanol	–	50	–	-At an electrode distance gap of 15 mm -Pulse duration of 400 ns -Treatment times of 3 and 9 min -Discharge voltages of 15 and 20 kV for argon -Discharge voltages of 20 and 25 kV for nitrogen -High voltage current of 30 mA	Using a treatment time of 9 min, 25% ethanol, and a discharge voltage of 25 kV for nitrogen	191.28 ± 7.12 mg GAE/g	Nutrizio et al. (2022)

### 4.1 Energy input

Extraction assisted by HVED is a cell disintegration technique (Nutrizio et al., 2020a). Cell disintegration index (Z) indicates the degree of cell damage caused by the energy input, and it evaluates the efficiency of the HVED treatment for the release of bioactive compounds such as polyphenols. A linear relationship exists between the Z value and TPC recovery (Barba et al., 2015a, Barba et al., 2015b). The degree of Z value increases as the treatment energy input rises (Boussetta and Vorobiev, 2014). The electrical conductivity is used to assess the Z value:

Z = (σ - σ_i_)/(σ_d_ - σ_i_)

Here, σ (μS/cm) represents the electrical conductivity of the samples, with the subscripts ‘i’ and ‘d’ indicating the conductivities of intact and fully damaged samples, respectively (Li et al., 2019). Also, Z values of 0 and 1 correspond to the intact and fully damaged cellular tissues, respectively (Hernández-Corroto et al., 2022).

According to Boussetta et al., 2012a, Boussetta et al., 2012b, while the optimum energy inputs for the highest extraction yield of polyphenols from grape stems and skins were 213 and 53 kJ/kg at laboratory scales, they were 400 and 133 kJ/kg at pilot scales, respectively (Boussetta et al., 2012b). Rajha et al. (2014) reported that the maximum polyphenol yield from vine shoots was achieved after HVED treatment with an energy input of 254 kJ/kg (Rajha et al., 2014). Moreover, according to Sarkis et al., 2015b, Sarkis et al., 2015a, when applying energy inputs of 0–291 kJ/kg to sesame cake, the maximum polyphenol content was achieved at the energy input of 83 kJ/kg (Sarkis et al., 2015a). Roselló-Soto et al. (2015) reported that when using HVED energy inputs of 0–109 kJ/kg and its subsequent solid-liquid extraction in 49% ethanolic solution, the most polyphenol content from olive kernels was recovered at an energy input of 66 kJ/kg (Roselló-Soto et al., 2015). One study revealed that the maximum TPC from grape pomace was achieved at an HVED energy input of 80 kJ/kg (Boussetta et al., 2011). Another study presented that the concentration of vine shoot polyphenols enhanced significantly from 100 to 110 mg/L at energy inputs of 304.8–609.5 kJ/kg (Rajha et al., 2015b). Moreover, Sarkis et al., 2015b, Sarkis et al., 2015a reported that increasing the HVED energy input from 40 to 240 kJ/kg led to an almost 66% increase in the recovery of polyphenols from sesame seeds (Sarkis et al., 2015b). According to El Kantar et al. (2018), increasing the energy input from 44 to 222 kJ/kg led to a 0.7% increase in the concentration of recovered polyphenols from orange peels (El Kantar et al., 2018). Hernández-Corroto et al. (2022) reported that the maximum concentration of polyphenols from pomegranate seeds was obtained at an energy input of 213 kJ/kg (Z value of 96%). However, applying an energy input of 107 kJ/kg (Z value of 72%) recovered only 50% of the polyphenols (Hernández-Corroto et al., 2022). Moreover, in one study, the highest Z value of 19.3% at an energy input of 609.5 kJ/kg recovered the maximum polyphenol content from vine shoots (Rajha et al., 2015b). El Kantar et al. (2019) reported that increasing the HVED energy input from 7.27 to 72 kJ/kg, increased the Z value from 0.36 to 0.72 and thus, improved the release of polyphenols from grapefruit peels (El Kantar et al., 2019). Similarly, N. Rajha et al., 2015a, Rajha et al., 2015b presented that increasing the HVED energy input from 49 to 242 kJ/kg resulted in about 1.5-fold higher recovery of polyphenols from vine shoots by increasing the Z value from 0.33 to 0.71 (Rajha et al., 2015a). These studies declared that the most TPC is obtained by increasing the Z value from 0.33 to 0.72.

### 4.2 Discharge voltage

Discharge voltage can also affect the recovery of polyphenols (Deng et al., 2018). According to Nutrizio et al., (2022), when applying two voltages of 15 and 20 kV for argon gas and also discharge voltages of 20 and 25 kV for nitrogen gas, the highest polyphenol concentration from oregano was obtained with nitrogen at 25 kV, treatment time of 9 min, and 25% ethanol as solvent (Nutrizio et al., 2022). Similarly, Nutrizio et al., 2020a, Nutrizio et al., 2020b reported that by using the same discharge voltages for argon and nitrogen gases, the most polyphenol content from wild thyme (*Thymus serpyllum* L.) was achieved with nitrogen at 20 kV, treatment time of 9 min, and 50% ethanol as solvent (Nutrizio et al., 2020b). Moreover, Žuntar et al. (2019) presented that with the same conditions as above, the maximum TPC from olive leaves was recovered with argon at 20 kV, treatment time of 9 min, and 50% ethanol. The obtained content was 3.2 times more than that of conventional extraction (Žuntar et al., 2019). Additionally, Nutrizio et al., 2020a, Nutrizio et al., 2020b declared that the highest polyphenol concentration from sage extracts (*Salvia officinalis* L.) was obtained with nitrogen gas at 25 kV, a treatment time of 9 min, and 33% ethanol, which was 1.8 fold more than the content obtained by conventional extraction (Nutrizio et al., 2020a). In one study, when using discharge voltages of 8, 11, 14, and 17 kV for the HVED treatment of spent coffee grounds for 20 min with a flow rate of 200 mL/min, a liquid-to-solid ratio of 50 mL/g, and 24% ethanolic solution, the maximum polyphenol content was achieved at the voltage of 11 kV. The recovered content was 20.03% more than that of conventional solvent extraction (Deng et al., 2018).

### 4.3 Electrode distance gap

Since the electrode distance gap is a determining factor for discharge formation, the recovery of polyphenols can be affected by the inter-electrode space (Boussetta et al., 2011). While large distances reduce the intensity of the electric field, short distances shorten the required time for the formation of electric arc discharge (Rodríguez De Luna et al., 2020). Furthermore, due to the incidence of arc discharge on numerous points of the needle at short distances, the discharge intensity of these points decreases at the same time (Boussetta et al., 2011). The HVED electrode distance gap is adjusted based on the conductivity of the sample and solvent (Barišić et al., 2020b; Rodríguez De Luna et al., 2020).

In one study, among HVED electrode distances of 3, 5, and 10 mm, the optimal value for the extraction of grape pomace TPC was 5 mm. This distance was also suitable for the better recovery of individual polyphenols (Boussetta et al., 2011). Xi et al. (2017) reported that using electrode distance gaps of 2, 3, 4, and 5 mm for the HVED-assisted extraction of polyphenols from pomegranate peel, the maximum value was obtained at the distance gap of 3 mm. However, increasing the distance to more than 3 mm reduced the recovery of polyphenols due to the weakened electric field (Xi et al., 2017). According to Deng et al. (2019), when applying electrode distance gaps of 3, 4, 5, and 6 mm for the HVED treatment of grape pomace, the most polyphenol content was recovered at the electrode distance of 4 mm (Deng et al., 2019). Moreover, Boussetta et al., 2012a, Boussetta et al., 2012b declared that an electrode distance of 5 mm was an optimal distance gap to recover polyphenols from grape seeds (Boussetta et al., 2012a). These studies declared that the optimal TPC is achieved at electrode distance gaps of 3–5 mm.

### 4.4 Type of solvent

Selecting an appropriate solvent is crucial in most solid-liquid extractions (Li et al., 2019). Generally, due to the advantages of low conductivity, approachability, and non-toxicity, water is the used solvent for HVED treatment (Deng et al., 2018). In one study, HVED pretreatment of grape pomace led to a ten times increment of aqueous extraction of polyphenols compared to the control (Boussetta et al., 2011). Similarly, Boussetta et al., 2009a, Boussetta et al., 2009b) declared the four times enhancement of aqueous extraction of grape skin polyphenols from HVED-treated samples than the control (Boussetta et al., 2009b). Žuntar et al. (2019) found that using water for the HVED-assisted extraction of polyphenols from olive leaves polyphenols recovered 2–6 times more TPC than aqueous conventional extraction (Žuntar et al., 2019).

Adding different ratios of ethanol can increase the effectiveness of the aqueous extraction of polyphenols through HVED. Since the dielectric constant of ethanol is lower than that of water, adding ethanol to an aqueous medium enhances the solubility of polyphenols by reducing the polarity of water (Bodoira et al., 2017; Boussetta et al., 2011). Moreover, ethanol accelerates the recovery of polyphenols by altering the phospholipid bilayer of cell membranes (Brianceau et al., 2016a, Brianceau et al., 2016b). Also, HVED-assisted extraction of polyphenols using ethanolic solutions destroys the bonds between polyphenols and plant material to which they are bound (Žuntar et al., 2019). It should be noted that by using high ethanolic concentrations in the treatment chamber, the conductivity is reduced and thus, the discharge may be impeded (Li et al., 2019). Besides, since most of the incoming energy is deposited into the solution and just a minor part can form a plasma channel, the discharge intensity decreases at high ethanolic concentrations, thus, more energy is required to create a plasma channel (Deng et al., 2019). According to Deng et al. (2018), among water and ethanolic solutions (20%, 35%, 50%, and 100%, v/v) used for the HVED-assisted extraction of polyphenols from spent coffee grounds, the discharge didn't occur at ethanolic solutions of 50% and 100%. However, water and ethanolic solutions of 20% and 35% were easier to discharge. They also declared that by enhancing the concentration of ethanol up to 24%, the recovery of polyphenols increased through HVED (Deng et al., 2018). In one study, among different ethanolic concentrations of 10, 20, 30, and 40% used for the HVED treatment of grape pomace, the maximum polyphenol yield was obtained at 30% ethanol (Deng et al., 2019).

There is also an optimal value above which enhancing the ethanol concentration does not increase the recovery of polyphenols during the diffusion process (Brianceau et al., 2016a, Brianceau et al., 2016b). In one study, HVED-assisted extraction of flaxseed cake polyphenols with 25% ethanol recovered 1.2–2.5 times more TPC than aqueous extraction (Boussetta et al., 2013b). Another study demonstrated that after HVED pretreatment of grape pomace, among water and ethanolic solutions of 10%, 20%, and 30% used for the subsequent diffusion process, 30% ethanol recovered the highest amount of phenolics (Boussetta et al., 2011). According to Brianceau et al. (2016a,b), among ethanolic concentrations of 0–50% used to extract TPC from the HVED-pretreated grape stems, the highest content was achieved at ethanol 44% (Brianceau et al., 2016a, Brianceau et al., 2016b). Moreover, Sarkis et al., 2015b, Sarkis et al., 2015a reported that among water and ethanolic solutions of 10%, 30%, and 50% used for the HVED-assisted extraction of sesame cake polyphenols for 60 min, 10% ethanol recovered the highest polyphenol content. This study also displayed that pretreatment with HVED can decrease the use of ethanol in the next extraction process (Sarkis et al., 2015a). In contrast, the extraction efficiency of polyphenols from HVED-pretreated samples is decreased in ethanolic concentrations of more than 50–60% (Boussetta et al., 2011).

Deep eutectic solvents (DESs) and aqueous glycerol can also be used for the extraction of polyphenols (El Kantar et al., 2019). DESs can increase the solubility of polyphenols by forming hydrogen bonds between the polyphenols and the DES molecules (Rente et al., 2021). In one study, among water, aqueous glycerol solutions (10%, 20%, and 30%), and DES of lactic acid: glucose used for the solid-liquid extraction of polyphenols from HVED-treated grapefruit peels, the highest polyphenol content was obtained by 20% glycerol solution, followed by DES of lactic acid: glucose (El Kantar et al., 2019).

### 4.5 Liquid-to-solid ratio

Liquid-to-solid ratio is a critical factor affecting the extraction efficiency of polyphenols (Deng et al., 2018). According to Barba et al., 2015a, Barba et al., 2015b, extraction at a higher solid-to-liquid ratio recovers more polyphenol content than at a lower solid-to-liquid ratio (Barba et al., 2015a, Barba et al., 2015b). Indeed, using a higher volume of the solvent led to a higher concentration gradient as the driving force of the extraction. Therefore, more solvent enters the cells and more polyphenols permeate to the solvent (Deng et al., 2019; Yan et al., 2018). However, by further increasing the liquid-to-solid ratio, the extraction efficiency of polyphenols reaches a plateau, and finally, the absorbed energy by the excess volume of solvent reduces the extractability of polyphenols (Deng et al., 2019; Xi et al., 2017). In addition, more energy will be consumed to remove the excess solvent from the obtained extract (Li et al., 2019). Boussetta et al. (2011) reported that among liquid-to-solid ratios of 2, 3, 5, 10, and 20 mL/gr used for the HVED-assisted extraction of grape pomace polyphenols, the polyphenol recovery enhances up to a liquid-to-solid ratio of 5 and then remains constant as this ratio is further increased (Boussetta et al., 2011). In one study, among liquid-to-solid ratios of 20, 30, 40, and 50 mL/gr, the most polyphenol content from pomegranate peel was achieved at the liquid-to-solid ratio of 30 mL/gr (Xi et al., 2017). Moreover, Deng et al. (2018) reported that among liquid-to-solid ratios of 30, 40, 50, and 60 mL/gr used for the HVED treatment of spent coffee grounds, the maximum polyphenol content was obtained at the ratio of 50 (Deng et al., 2018). In another study, after HVED treatment of rapeseed at liquid-to-solid ratios of 5–20 mL/gr, the maximum polyphenol content was obtained at the ratio of 20 (Barba et al., 2015a, Barba et al., 2015b). According to Deng et al. (2019), among liquid-to-solid ratios of 30, 40, 50, and 60 mL/gr used for the recovery of grape pomace polyphenols through HVED treatment, the maximum polyphenol content was obtained at the ratio of 40 (Deng et al., 2019). Banožic et al. (2021) presented that when using liquid-to-solid ratios of 300, 500, and 700 mL/g for the HVED-assisted extraction of TPC from tobacco waste, the highest value was achieved at the ratio of 700 mL/g (Banožić et al., 2021). Moreover, Hernández-Corroto et al. (2022) reported that among liquid-to-solid ratios of 4–19 mL/gr, HVED treatment at the optimum ratio of 9 recovered 78% of polyphenols (Hernández-Corroto et al., 2022).

### 4.6 pH of dissolution

pH of solvent may also influence the extractability of polyphenols through alterations in their solubility and interactions within plant substances (Parniakov et al., 2015). The pH of the solvent can be adjusted with mother solutions of hydrochloric acid (C_HCL_ = 1 M), potassium hydroxide (C_KOH_ = 1 N), or sodium hydroxide (C_NaOH_ = 2 M) (Brianceau et al., 2016a, Brianceau et al., 2016b; Parniakov et al., 2014). Using maximal concentrations of 0.07% HCl and 0.04% KOH in the solvent can limit the acidic and alkaline hydrolyses of polyphenols (Brianceau et al., 2016a, Brianceau et al., 2016b).

Generally, acidic solvents are suitable for the high recovery of most polyphenols (Brianceau et al., 2016a, Brianceau et al., 2016b). While flavonols are better recovered in a basic medium (pH = 8.5), the higher extractability of flavan-3-ols and stilbenes is obtained at an acidic condition (pH = 2.5) (Li et al., 2019). Moreover, acidic and basic conditions are more efficient than intermediate pH values for extracting piceid and resveratrol (Brianceau et al., 2016a, Brianceau et al., 2016b). In one study, after HVED pretreatment of the olive kernel at an energy input of 55 kJ/kg followed by extraction at 25% ethanol, the maximum polyphenol content was obtained at pH values of 2.5 and 12. However, when using the same HVED energy input and ethanolic concentration of 50%, the optimum polyphenol content was obtained at pH 2.5 (Roselló-Soto et al., 2015). Parniakov et al. (2014) reported that when using acidic (pH = 2.5), neutral (pH = 7), and alkaline (pH = 11) conditions for the HVED-assisted extraction of polyphenols from papaya peels, the maximum phenolic yield was obtained at acidic condition (Parniakov et al., 2014). This can be due to the degradation of plant material in the presence of acid, increasing the extractability and solubility of polyphenols (Brianceau et al., 2016a, Brianceau et al., 2016b). Moreover, this acidic condition results in the formation of hydrolysis products that react with the Folin–Ciocalteu reagent and increase the TPC. Additionally, acidic pH can increase phenolics' extractability by dissociating non-covalent bindings of some polyphenols to proteins (Roselló-Soto et al., 2015).

The high extraction efficiency of polyphenols at pH = 12 is due to the alkaline hydrolysis of esteric bonds between polyphenols, which increases the solubility of polyphenols through the dissociation of the phenolic hydroxyl groups (Li et al., 2019; Roselló-Soto et al., 2015). It should be noted that higher pH values may also cause the oxidation of polyphenols (Li et al., 2019). In one study, among alkaline solution (0.1 M NaOH) and 20% acid-based DESs (choline chloride: citric acid, choline chloride: acetic acid, and choline chloride: lactic acid) used for the solid-liquid extraction of polyphenols from HVED-pretreated pomegranate seeds, NaOH solution resulted in almost absolute recovery of polyphenols compared to DESs (Hernández-Corroto et al., 2022).

### 4.7 Extraction time and temperature

Since the value of the Z disintegration index increases with the duration of discharges, extraction time is one significant factor in the recovery of TPC (Deng et al., 2018; El Kantar et al., 2018). While too long HVED treatment time leads to needless energy input, too little treatment time may be insufficient for the recovery of phenolics (Deng et al., 2018). It should also be noted that HVED pretreatment and its subsequent diffusion process use lower extraction time and temperature than the extraction without pretreatment (Boussetta et al., 2013a).

According to Brianceau et al. (2016a,b), when using HVED treatment times of 0–4 ms, the maximum TPC from grape stems was obtained at 3.6 ms (Brianceau et al., 2016a, Brianceau et al., 2016b). Moreover, Boussetta et al., (2011) reported that among HVED treatment times of 0.05, 0.5, 0.8, 1, 5, and 10 ms, the most grape pomace polyphenol content was achieved at 0.8 ms. They also announced that in the first 30 min of subsequent solid-liquid extraction, polyphenols were increasingly released from the plant material into the solvent. Then, the threshold was achieved and the extraction kinetic was reduced from 30 to 60 min (Boussetta et al., 2011). Similarly, Xi et al. (2017) reported that the highest extraction efficiency of polyphenols from pomegranate peel was obtained after 30 min of HVED-assisted diffusion, which was 1.2-fold more than the amount recovered by maceration for 60 min (Xi et al., 2017). In one study, for the HVED-assisted extraction of flaxseed cake polyphenols, the effective diffusion times for the aqueous and hydro-ethanolic extractions were about 37 and 26 min, respectively (Boussetta et al., 2013b). Moreover, Deng et al. (2018) reported that among HVED-assisted diffusion times of 10, 15, 20, and 25 min used for the extraction of polyphenols from spent coffee grounds, the maximum polyphenol content was achieved after 20 min of extraction, which was 20.03% more than the content obtained by 150 min of conventional solvent extraction (Deng et al., 2018). According to Boussetta et al., 2009a, Boussetta et al., 2009b, the highest concentration of polyphenols from HVED-treated grape skin was obtained after 1 h of subsequent diffusion procedure (Boussetta et al., 2009b). Moreover, according to Sarkis et al., 2015b, Sarkis et al., 2015a, aqueous diffusion of HVED-treated sesame cake for 10 min recovered 3.2 times more polyphenols than the control (Sarkis et al., 2015a). Xi et al. (2017) reported that HVED treatment of pomegranate peel for 30 min recovered 23.78% more polyphenol content than warm water maceration for 60 min (Xi et al., 2017). In one study, HVED-assisted diffusion of fresh grape pomace for 1 h increased the polyphenol content by 30%. However, the same content was not achieved even after 4 h of extraction without the use of HVED (Boussetta et al., 2009a). In another study, HVED-assisted extraction of polyphenols from grape seeds reduced the effective diffusion time by 4.6 times compared to the control (Boussetta et al., 2012a).

Diffusion temperature can also affect the extraction efficiency of polyphenols (Antony and Farid, 2022). Heating increases the extraction efficiency of polyphenols by altering the cell membranes and enhancing cell permeability (Boussetta et al., 2009a). Moreover, it can facilitate the diffusion of polyphenols to the solvent by weakening the phenol–protein interactions in plant material (Sarkis et al., 2015a). Using moderate temperatures during HVED-assisted extraction is a prerequisite to increase the recovery of phenolics (Boussetta et al., 2011; Sarkis et al., 2015a). In one study, among temperatures of 20–60 °C used for the solid-liquid extraction of polyphenols from HVED-treated flaxseed cake, 60 °C was the most efficient temperature for the maximum recovery of phenolics (Boussetta et al., 2013b). In another study, when using temperatures of 20, 40, and 60 °C for HVED-assisted extraction of sesame cake polyphenols, the optimum polyphenol content was obtained at 60 °C (Sarkis et al., 2015a). Furthermore, Boussetta et al. (2011) reported that increasing the diffusion temperature from 20 to 60 °C led to a 33% increase in the grape pomace polyphenol content in HVED-treated samples (Boussetta et al., 2011). They also found that this increase in temperature recovered more polyphenols from HVED-treated grape skins than untreated ones (Boussetta et al., 2009b). According to El Kantar et al. (2018), hydro-ethanolic diffusion of HVED-treated orange peels at 50 °C for 1 h led to 1.72 times more polyphenol content than untreated samples (El Kantar et al., 2018).

## 5 Factors influencing the stability of polyphenols

Since polyphenols are prone to degradation with minor changes in the extraction process, extracting these bioactive compounds can be challenging. The degradation mechanism of polyphenols during the HVED treatment may be due to the formation of intermediate oxidative products. The reaction of hydroxyl radical with phenolics leads to the formation of phenoxyl radical. The phenoxyl radical can also be generated directly through the UV light produced during the electric discharges. The subsequent reaction of phenoxyl radical with hydroxyl radical or oxygen forms pyrocatechol, hydroquinone, resorcinol, and 1,4-benzoquinone (El Kantar et al., 2018). Factors like light, temperature, and oxygen may also affect the stability of polyphenols during the treatment and storage time (Sridhar et al., 2021). In this regard, covering the treatment chamber with aluminum foil is essential to prevent the degradation of polyphenols through the air or light (Rajha et al., 2015a). Moreover, the recovered phenolic extract should be stored in a dark place at 4 °C until its use (Deng et al., 2018). In the presence of oxygen, polyphenols are oxidized to quinones by enzymatic browning. Polyphenols may also be unstable through autoxidation in the presence of oxygen. Another instability mechanism is the epimerization of polyphenols at high temperatures, pH, or in the presence of metal ions (Cao et al., 2021). The effect of HVED processing parameters on the stability of polyphenols is reviewed here.

### 5.1 Energy input

High HVED energy input forms radical species that can increase the degradation of polyphenols through oxidation (Boussetta and Vorobiev, 2014). The dielectric breakdown during the HVED treatment generates high-energy electrons, dissociating water molecules into hydroxyl and hydrogen radicals. The reaction of these reactive species with each other or other molecules leads to the formation of hydrogen peroxide (H_2_O_2_) (Mededovic and Locke, 2007). Boussetta et al. (2011) reported that energy inputs ranging from 80 to 800 kJ/kg degraded the polyphenols recovered from grape pomace (Boussetta et al., 2011). According to Roselló-Soto et al., (2015), HVED energy inputs of more than 100 kJ/kg damaged the TPC from the olive kernel (Roselló-Soto et al., 2015). El Kantar et al. (2018) reported that applying energy inputs above 222 kJ/kg reduced the polyphenols yield by 0.05% due to the formation of oxidizing species (El Kantar et al., 2018). Moreover, in one study, increasing the HVED energy input from 406.3 to 609.5 kJ/kg increased the H_2_O_2_ concentration of the extracts from 1 to 5 mg/L. However, since increasing the energy input led to a 25-fold higher concentration of vine shoot polyphenols than that of H_2_O_2_, the antioxidant properties of polyphenols prevented H_2_O_2_ formation. Thus, no degradation of polyphenols by HVED was observed (Rajha et al., 2015b). It should be noted that the antiradical effect of polyphenols is concentration-dependent (Vladimir-Knežević et al., 2011).

### 5.2 Discharge voltage

Although the recovery of polyphenols increases with increasing the applied voltage, they may also be degraded by extremely high voltages (Deng et al., 2018). Indeed, using too high voltages leads to the formation of species such as hydroxyl radicals, ozone, and atomic hydrogen which can damage the recovered polyphenols through oxidation (Nutrizio et al., 2020a). In one study, increasing the electrical discharges up to 25 kV led to the generation of additional reactive species and the deterioration of polyphenols from olive leaves extracts (*Olea europaea* L.) (Žuntar et al., 2019). Moreover, Yan et al. (2018) reported that increasing the electric discharge to more than 13 kV led to the degradation of polyphenols from peanut shells (Yan et al., 2018). According to Deng et al. (2018), increasing the discharge voltage from 11 to 17 kV led to the degradation of polyphenols from spent coffee grounds (Deng et al., 2018).

### 5.3 Electrode distance gap

Too small electrode distance gaps may cause high electric field intensities affecting the structure of polyphenols (Li et al., 2019). Indeed, the generated high plasma energy density between narrow electrode gaps damages most of the polyphenols by depositing more energy into the solution (Cheng et al., 2012). According to Xi et al. (2017), decreasing the electrode distance gap to less than 3.1 mm led to the rapid reduction of the recovered polyphenols from pomegranate peels (Xi et al., 2017). In one study, applying an electrode distance gap of 3 mm for the HVED treatment of grape pomace resulted in an intensive discharge and thus, degradation of polyphenols (Deng et al., 2019).

### 5.4 pH of dissolution

The solvent's initial pH influences the degradation of polyphenols during the HVED-assisted extraction (El Kantar et al., 2018). Indeed, the formation of reactive species during discharge application may degrade polyphenols depending on the pH (Brianceau et al., 2016a, Brianceau et al., 2016b). HVED treatment leads to the generation of ozone molecules, which react with water to form hydrogen peroxide. Then hydrogen peroxide is decomposed to hydroxyl radicals. HVED treatment at high pH values degrades polyphenols due to the faster decomposition of ozone molecules and thus, increased amount of degradative hydroxyl radicals. On the other hand, ozone molecules are stable during the HVED treatment at low pH values (Ershov and Morozov, 2008). Generally, flavonoids are degraded in neutral and alkaline solutions (Boussetta et al., 2011). Moreover, the degradation of catechin and epicatechin increases at high pH values (El Kantar et al., 2018). While these two classes of polyphenols are stable at pH ranges of 2–5, they are degraded in several minutes by increasing the pH value to more than 8 (Boussetta et al., 2011). Acidic solvents may also limit the oxidation of flavan-3-ols and protect them during electric discharges (Brianceau et al., 2016a, Brianceau et al., 2016b). According to Roselló-Soto et al. (2015), the maximum recovery of olive kernel polyphenols at pH = 2.5 was due to the better stability of phenolics at highly acidic conditions (Roselló-Soto et al., 2015). Brianceau et al. (2016a,b) found that when using pH values of 2.5, 5.5, and 8.5 for the HVED-assisted extraction of polyphenols from grape stems, a pH of 2.5 had a protective effect on polyphenols during electric discharges (Brianceau et al., 2016a, Brianceau et al., 2016b). Moreover, Boussetta et al. (2011) reported that since the pH of grape pomace suspension was 4, polyphenols were stable during the HVED treatment (Boussetta et al., 2011).

### 5.5 Extraction time and temperature

An optimal HVED treatment time should be accurately selected, as excessive extraction time may cause the deterioration of polyphenols (Brianceau et al., 2016a, Brianceau et al., 2016b). In one study, increasing the HVED-assisted diffusion time from 20 to 25 min reduced the recovered polyphenol content from spent coffee grounds (Deng et al., 2018). According to Xi et al. (2017), increasing the HVED diffusion time to more than 31 min decreased the extracted polyphenols from pomegranate peel (Xi et al., 2017). Moreover, Brianceau et al. (2016a,b) reported that increasing the HVED treatment time to more than 3.6 ms reduced the obtained TPC from grape stems. This study also indicated that applying long HVED treatment time at a high pH value led to the loss of flavan-3-ols (Brianceau et al., 2016a, Brianceau et al., 2016b).

The electrically induced damages of HVED on cellular tissues are more significant at higher temperatures (Boussetta et al., 2009a). However, due to the oxidation of polyphenols at temperatures above 60 °C, this should be considered the upper limit to prevent the thermal degradation of phenolics (Boussetta et al., 2011). In one study, a temperature of 60 °C was used as the maximum temperature for the HVED-assisted extraction of polyphenols from grape seeds (Liu et al., 2011). Sarkis et al., 2015b, Sarkis et al., 2015a affirmed that no degradation of polyphenols occurred during the solid-liquid extraction of polyphenols from HVED-pretreated sesame cake at temperatures 0–60 °C (Sarkis et al., 2015a).

## 6 Comparison of HVED with novel extraction techniques of PEF and US

This paper compares HVED, PEF, and US novel extraction techniques, focusing on the specific equipment used, extraction and stabilization efficiencies of polyphenols, energy consumption and operating costs, environmental impacts, and potential limitations. Moreover, Table 2 presents the HVED, PEF, and US-assisted extraction of polyphenols under various experimental conditions.

**Table 2:** The HVED, PEF, and US-assisted extraction of polyphenols under various experimental conditions.

Category	Source	Extraction technique	Experimental conditions		Results	References
			Common conditions	Other conditions		
**Fruits and their by-products**	Pomegranate peels	US	Extraction using hot water at a liquid-to-solid ratio of 1:10 at the temperature of 50 °C	- Power of 400 W -Frequency of 24 kHz	-The highest polyphenol concentration was obtained with HVED (46 ± 0.5 mg GAE/g DM), followed by PEF (39 ± 2 mg GAE/g DM), and US (14.5 ± 0.8 mg GAE/g DM) after 7 min -The higher recovery of ellagic acid with PEF than with HVED and US techniques -The higher recovery of gallic acid with HVED than with PEF and US techniques -No degradative effects of these three techniques on polyphenols	Rajha et al. (2019)
		PEF		-Electrode distance gap of 4 cm -Voltage of 40 kV -Electric field of 10 kV/cm		
		HVED		-Electrode distance gap of 40 mm -Voltage of 40 kV -Electric field of 10 kV/cm		
	Grape by-Product	US	Extraction using 50% (v/v) ethanol acidified with 1% (v/v) HCl, and 50% (v/v) methanol acidified with 1% (v/v) HCl at a liquid-to-solid ratio of 50 for 5, 10, and 15 min	-Frequency of 35 kHz -Temperatures of 20, 40, and 80 °C	HVED treatment using ethanol-based solvent, frequency of 100 Hz, and electric field intensity of 60 kV/cm for 15 min recovered more TPC (3023.57 mg GAE/100 g DM) than the US treatment at 80 °C for 15 min (604.42 mg GAE/100 g DM)	Lončarić et al. (2020)
		HVED		-Electrode distance gap of 5 mm -Voltage of 30 kV -Frequencies of 20, 50, and 100 Hz -At room temperature		
	Vine shoots	US	-Extraction using water at a liquid-to-solid ratio of 10 at the temperature of 50 °C -In the subsequent extraction process: using ethanol at a liquid-to-solid ratio of 20 at the temperature of 50 °C	-Maximal power of 400 W -Maximal frequency of 24 kHz -Treatment energy of 4364 kJ/kg	-The higher efficiency of HVED than PEF and US techniques for the extraction of polyphenols -US (Z value = 0.2, Energy = 4364 kJ/kg), PEF (Z value = 0.61, Energy = 484 kJ/kg), and HVED (Z value = 0.71, Energy = 242 kJ/kg) treatments recovered 0.14, 0.26, and 0.3 mg/mL polyphenols, respectively	(Barba et al., 2015a, Barba et al., 2015b)
		PEF		-Electrode distance gap of 3 cm -Electric field strength of 13.3 kV/cm -Energy input of 484 kJ/kg -Frequency of 0.5 Hz		
		HVED		-Electrode distance gap of 5 mm -Voltage of 40 kV -Energy input of 242 kJ/kg -Frequency of 0.5 Hz		
	Grape pomace	US	Extraction at a liquid-to-solid ratio of 10 (v/w)	-Maximal power of 400 W -Frequency of 24 kHz -Amplitude of 100% -Energy consumption of 0–2727 kJ/kg	HVED, PEF, and US techniques recovered 88%, 47%, and 44% of TPC, respectively	Rajha et al. (2015a)
		PEF		-Frequency of 0.5 Hz -Electrode distance gap of 3 cm -Electric field strength of 13.3 kV/cm -Energy input of 0–564 kJ/kg		
		HVED		-Electrode distance gap of 1.25 cm -Voltage of 40 kV -Frequency of 0.5 Hz -Energy input of 0–218 kJ/kg		
**Oilseed crops and their by-products**	Sesame cake	PEF	-Extraction using water at a liquid-to-solid ratio of 8.5, energy input of 0–291 kJ/kg, frequency of 0.5 Hz, and treatment time of 1–7 ms -In the subsequent extraction process: using water and hydroethanolic solutions of 10%, 30%, and 50% at a liquid-to-solid ratio of 20 at the temperatures of 20, 40, and 60 °C	-Electrode distance gap of 3 cm -Electric field of 13.3 kV/cm	-HVED caused the permeabilization of most of the cell membranes and disruption of most of the cell walls compared to PEF treatment -The higher efficiency of HVED than the PEF technique for the aqueous extraction of polyphenols -Similar efficiencies of HVED and PEF techniques for hydroethanolic extraction of polyphenols	Sarkis et al. (2015a)
		HVED		-Electrode distance gap of 5 mm		
	Sesame seeds	PEF	-Covering sesame in water overnight and then extraction at a liquid-to-solid ratio of 3, voltage of 40 kV, frequency of 0.5 Hz, and different energy inputs of 40, 80, 160, and 240 kJ/kg	-Electrode distance gap of 2 cm -Electric field strength of 20 kV/cm	HVED recovered more polyphenols than the PEF technique in all applied energy inputs	Sarkis et al. (2015b)
		HVED		-Electrode distance gap of 5 mm		
	Olive kernel	US	-Extraction using water at a liquid-to-solid ratio of 5 -In the subsequent extraction process: using ethanolic solutions of 0–50 % (v/v) at a liquid-to-solid ratio of 10 and temperature of 20 °C	-Power of 400 W -Frequency of 24 kHz -Amplitude of 100 %	The higher extraction efficiency of polyphenols using HVED (255 mg GAE/L) compared to PEF (146 mg GAE/L) and US (140 mg GAE/L) techniques/Faster extraction kinetics using HVED compared to US and PEF techniques	Roselló-Soto et al. (2015)
		PEF		-Electrode distance gap of 3 cm -Electric field strength of 13.3 kV/cm Frequency of 0.5 Hz -Energy inputs of 0–109 kJ/kg -Treatment time of 4 ms		
		HVED		-Electrode distance gap of 5 mm -Voltage of 40 kV -Energy inputs of 0–109 kJ/kg -Treatment time of 4 ms		

### 6.1 Specific equipment used

The HVED extraction systems are equipped with two stainless-steel electrodes (Barišić et al., 2020a). While one of the electrodes is connected to the high-voltage generator, the other is attached to the ground (Ranjha et al., 2021). The batch HVED extraction system contains a treatment chamber and a high voltage pulse generator providing 40 kV-10 kA discharges (Fig. 3a) (Chadni et al., 2019). The continuous HVED extraction system comprises a high voltage pulse generator, an oscilloscope, a treatment chamber, a cooling system, a peristaltic pump, and voltage and current measuring devices. The high voltage generator provides frequencies of up to 100 Hz and 20 kV–10 kA discharges for a few microseconds (Li et al., 2019). The peristaltic pump provides the flowing power and the oscilloscope is for data acquisition (Deng et al., 2019) (Fig. 3b & 3.c). Moreover, the circulating HVED extraction system consists of a high voltage pulse generator, a treatment chamber, an extraction tank, and a transport unit. The high voltage pulse generator provides frequencies of up to 50 Hz and voltages of up to 40 kV. The extraction tank containing an agitator is applied for diffusion. The pump and pipeline of the transport unit provide the power and pathway for circulating flow (Fig. 3d) (Li et al., 2019).

**Fig. 4:**
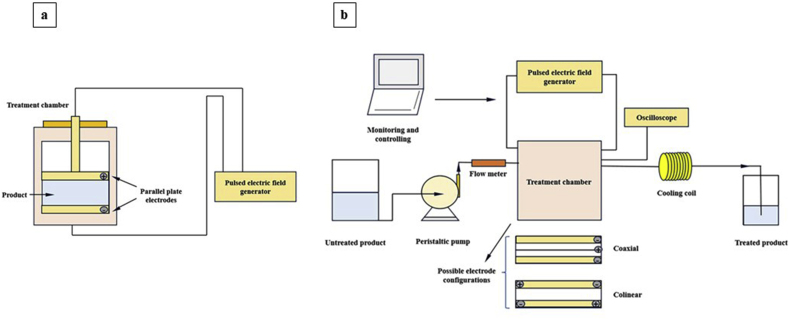
Schematics of batch (a) and continuous (b) PEF extraction systems.

The PEF extraction system can be classified into batch and continuous systems. The batch PEF extraction system consists of a treatment chamber and equipment for solid-liquid extraction. The treatment chamber is a polypropylene cylinder with two parallel stainless electrodes. At first, the sample is treated with a small volume of solvent between the electrodes of the treatment chamber connected to a PEF generator. The obtained treated sample is then stirred on a magnetic agitator for the subsequent solid-liquid extraction (Fig. 4a) (Ranjha et al., 2021). On the other hand, the continuous PEF extraction system comprises a high voltage pulse generator, a treatment chamber, a product handling system, and a set of monitoring equipment. The treatment chamber contains coaxial and colinear electrodes. The oscilloscope indicates the output voltage (up to 40 kV) and the cooling coil avoids the temperature rise during the treatment. The sample is pumped to the treatment chamber through a peristaltic pump (Fig. 4b) (Xi et al., 2021).

**Fig. 5:**
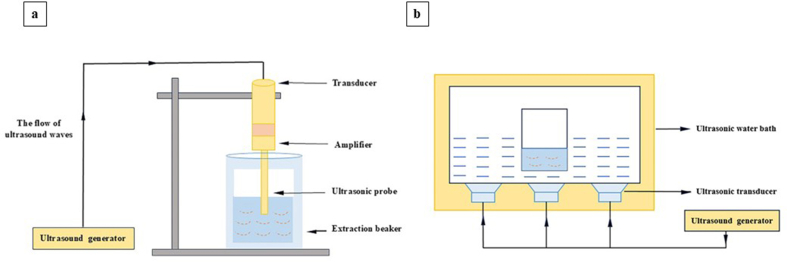
Schematics of probe (a) and bath (b) US extraction systems.

Two types of US extraction systems are available in the food industry; ultrasonic water baths and probes. The probe system consists of a US generator, a transducer, an amplifier, and a probe. The transducer converts the high-frequency electrical energy produced by the US generator to mechanical energy. The amplifier intensifies the mechanical energy and then, the probe distributes the acoustic waves into the solution (Fig. 5a). The bath system also contains a US generator, transducers, and a bath. The transducers distribute the acoustic waves into the bath (Fig. 5b). Unlike the probe system, samples are not exposed to the direct waves in the bath system (Strieder et al., 2019).

### 6.2 Extraction and stabilization efficiencies of polyphenols

The efficiency of novel extraction techniques can be compared through cellular disintegration resulting from the phenomenon occurring in each technique (Boateng and Clark, 2024). The shock waves, turbulence, and shear stress caused by the generation and collapsing of bubbles (cavitation phenomenon) in the US technique disrupt the plant cell walls and membranes, thereby increasing the extraction efficiency of bioactive compounds (Dukić et al., 2023). The PEF technique facilitates the extractability of target compounds through a high transmembrane potential difference that forms irreversible or temporary pores on the plant cell walls and membranes (electroporation phenomenon). Electroporation occurs at electric fields greater than 0.5 V/nm, based on the lipid bilayers of the cell membrane (Boateng and Clark, 2024). Similarly, the HVED technique affects the cell walls and membranes through the electroporation effect (Nutrizio et al., 2020a). Moreover, the arc formed during electrical discharges leads to more cellular disintegration and higher efficiency of HVED than the US and PEF novel extraction techniques (Li et al., 2019; Sarkis et al., 2015b). According to Rajha et al., (2019), HVED-assisted extraction of polyphenols from pomegranate peels recovered 3 and 1.3 times more phenolics than the US and PEF techniques, respectively (Rajha et al., 2019). Similarly, Roselló-Soto et al. (2015) found that, at equivalent energy inputs, HVED extracted approximately 1.8 and 1.7 times more phenolic compounds from the olive kernel than US and PEF technologies, respectively (Roselló-Soto et al., 2015). In one study, while a maximum cell damage of 67% was obtained after PEF treatment of the sesame cake with an energy input of 125 kJ/kg, a maximum cell damage of 90% was achieved after applying an HVED energy input of 83 kJ/kg to these samples. This study showed that at a fixed energy input of 83 kJ/kg, HVED was more efficient than PEF treatment for the aqueous extraction of polyphenols from sesame cake. However, the extraction efficiency of both electrical treatments was very similar using ethanolic solutions of 10%, 30%, and 50% (Sarkis et al., 2015a). Another study presented that at Z values above 0.4, HVED recovered more than two times polyphenols from grape pomace compared to the US and PEF techniques, with lower energy inputs (Barba et al., 2015a, Barba et al., 2015b). Moreover, Lončarić et al. (2020) found that HVED-assisted extraction of polyphenols from grape by-products recovered 5-fold more polyphenol content than the US technique (Lončarić et al., 2020). Sarkis et al., 2015b, Sarkis et al., 2015a also reported that HVED extracted approximately 2 times more polyphenols from sesame seeds than the PEF technology (Sarkis et al., 2015b).

Since the extraction assisted by the US, PEF, and HVED techniques is efficient at room temperature or slightly elevated temperatures, these techniques prevent the thermal degradation of thermolabile compounds such as polyphenols (Banožić et al., 2021). Compared to other extraction techniques, UAE recovers polyphenols with minimal breakdown of these compounds (Alara et al., 2021). Rajha et al., (2019) declared that none of the HVED, PEF, and US techniques had degradative effects on the recovered polyphenols from pomegranate peels (Rajha et al., 2019). On the other hand, in one study, the configuration of extracted lignans was changed due to the reaction of these substances with active species produced during the HVED treatment (Sarkis et al., 2015a). In contrast, Sarkis et al., 2015b, Sarkis et al., 2015a reported that these active species did not change the lignan profile of the extracts from sesame seeds (Sarkis et al., 2015b).

### 6.3 Energy consumption and operating costs

The economic assessment of each extraction process relies on the plant material, solvent, equipment, and energy consumption (Nutrizio et al., 2022). HVED consumes less energy than the PEF and US techniques for recovering polyphenols (Barba et al., 2015a, Barba et al., 2015b). Moreover, these novel techniques are more energy-efficient than the conventional solvent extraction methods (Rajha et al., 2015a). Barba et al., 2015a, Barba et al., 2015b reported that the energy required for HVED-assisted extraction of 1 mg of TPC from grape pomace remained almost stable at different Z values. In contrast, increasing the Z value led to more energy consumption by the US and PEF techniques to extract 1 mg of TPC. Although a high Z value is necessary to improve the recovery of bioactive compounds, it can increase the costs for the PEF and US techniques (Barba et al., 2015a, Barba et al., 2015b). On the other hand, since PEF requires less powerful equipment than HVED at an industrial scale, the equipment cost of the PEF is lower than the HVED technique. In 2016, the estimated prices to improve the industrial extraction processes ranged from 75,000 to 400,000 € for the PEF and from 100,000 to 500,000 € for the HVED equipment, depending on the costs of the treatment chamber, monitoring systems, and the manufacturer's profit margin (Puértolas and Barba, 2016). Indeed, although the HVED technique has a low operational cost regarding electricity consumption in the long term (i.e., 90 kW/h × 0.05 $/kW/h = 4.5 $/h), it is an expensive technique in terms of equipment cost (Žuntar et al., 2019).

### 6.4 Environmental impacts

US, PEF, and HVED are environmentally friendly extraction processes that apply green solvents for extracting polyphenols (Rajha et al., 2015a). These techniques are accompanied by low CO_2_ emissions and little environmental impact (Jara-Quijada et al., 2024; Nutrizio et al., 2022). Nutrizio et al. (2022) reported that HVED-assisted extraction of polyphenols from oregano had a significantly lower CO_2_ emission than conventional extraction methods such as infusion and maceration. While 1 min of HVED extraction had the lowest environmental impact, extraction by infusion showed the highest impact (Nutrizio et al., 2022). Jara-Quijada et al. (2024) compared the environmental impacts of extractions assisted by PEF, US, and microwave techniques to recover polyphenols from green tea. The results showed that compared to the PEF technique, the US and microwave techniques generated about 16 and 12 times more CO_2_ emissions, respectively. Indeed, the PEF technique had a considerably lower environmental impact than these two techniques (Jara-Quijada et al., 2024).

### 6.5 Potential limitations

The free radical species produced during the HVED, PEF, and US treatments may hydroxylate the polyphenols, and form hydroxyl ring carbonyl radicals capable of setting off a reaction that can damage phenolics (Liu et al., 2024). Since the HVED technique causes severe damage to the cell structure, it is less selective than the US and PEF techniques for extracting target compounds (Li et al., 2019). This can enhance the economic cost of the process due to the difficult purification of the recovered extracts (Puértolas and Barba, 2016). Moreover, the HVED technique needs precise control of the energy input (Carpentieri et al., 2021).

The limitations of the PEF technique include high equipment costs and challenges in treating conductive materials (Carpentieri et al., 2021). Additionally, there is a risk of the corrosion and ingress of the electrode material into the food products (Eyshi et al., 2024). Bubbles and gases generated during the PEF treatment may lead to operational issues and non-uniform heating (Arshad et al., 2020). Moreover, increasing energy consumption causes more CO_2_ emissions (Boateng and Clark, 2024). The lack of practical electrical systems and economic studies limits the industrial scaling up of this process. There are also no adequate protocols for the PEF treatment of food products (Arshad et al., 2020).

The US technique has limitations such as non-selective extraction and low treatment efficiency in viscous systems (Koina et al., 2023). This technique requires the subsequent separation and purification steps (Carpentieri et al., 2021). In the ultrasonic bath system, water absorbs some energy and reduces the energy reaching the sample (Boateng and Clark, 2024). Moreover, the low power density of this system prolongs the extraction time (Fernandes and Rodrigues, 2023). Although the ultrasonic probe system has a higher power density than the bath one, it may contaminate the food products with metals released from the probe (Bernardi et al., 2021; Fernandes and Rodrigues, 2023). Besides, this expensive extraction technique is suitable for processing low amounts of samples (Boateng and Clark, 2024).

## 7 Future aspects

Since the HVED uses green solvents such as water and ethanol for extraction, it is considered harmless to the environment (Alirezalu et al., 2020). Decontamination of food, disinfection of water, inactivation of enzymes, and extraction of bioactive compounds from plant materials are some of the wide applications of this technology (Dukić et al., 2023). However, the HVED technique still has certain challenges that should be addressed for its broader implementation in the future: 1) One of the main challenges of HVED is the possibility of implementing this technique on an industrial scale (Li et al., 2020). Since a higher energy input is needed at the pilot scale to achieve the same yields as the laboratory scale, enlarging the process variables to obtain the same yield at the industrial scale is not feasible (Li et al., 2019). Thus, it is crucial to elucidate the mass transfer dynamics of HVED to scale up the industrial process (Li et al., 2020). The industrial scaling up of this technique requires continuous and circulating systems that enable the extraction process in continuous conditions (Li et al., 2019). Moreover, to make the industrialization process of the HVED technique profitable, the added value should be high enough to compensate for the equipment and electricity costs (Puértolas and Barba, 2016). 2) Despite the high efficiency of HVED-assisted extraction, its utilization in the food industry may be decreased due to the possible contamination of the treated sample by electrolysis by-products and free radicals (Parniakov et al., 2014). These free radicals, mainly reactive oxygen species (ROS) and reactive nitrogen species (RNS) can oxidize the target molecules and form other secondary metabolites that are harmful to the consumer (Boateng and Clark, 2024). The ROS molecules can also damage the body's nucleic acids, proteins, and lipids leading to several chronic and degenerative diseases (Martemucci et al., 2022). Therefore, the energy input of the HVED technique should be controlled to prevent the generation of too many free radicals (Li et al., 2019).

## 8 Conclusion

The existence of polyphenols within the plant cell membranes and vacuoles complicates their efficient recovery and later application. Therefore, it is crucial to select a suitable extraction technique. The application of conventional solvent extraction methods has been limited because of their hazardous organic solvents. Substituting these extraction methods with the innovative HVED technique offers several advantages, including less extraction time, lower processing temperature, and little solvent consumption. This review implies that the extraction efficiency of polyphenols can be increased by optimizing the HVED processing parameters. Moreover, acidification and other processing parameters may prevent the oxidation of polyphenols. The HVED technique recovers more polyphenols than the conventional solvent extraction, US, and PEF methods. It is an environmentally friendly technique that enables the extraction of polyphenols with lower energy consumption than other methods.

## CRediT authorship contribution statement

**Leila Abbaspour:** Data curation, Investigation, Writing – original draft. **Nazila Ghareaghajlou:** Data curation, Investigation, Writing – original draft. **Mohammad Reza Afshar Mogaddam:** Investigation, Supervision, Writing – review & editing. **Zahra Ghasempour:** Conceptualization, Supervision, Project administration, Writing – review & editing.

## Funding

This work is financially supported by the Tabriz University of Medical Sciences (Grant number: 71242; Ethics Code: IR.TBZMED.VCR.REC.1401.387).

## Declaration of competing interest

The authors declare that they have no known competing financial interests or personal relationships that could have appeared to influence the work reported in this paper.

## Data Availability

No data was used for the research described in the article.
